# Oxidative Stress and Inflammation Interdependence in Multiple Sclerosis

**DOI:** 10.3390/jcm8111815

**Published:** 2019-11-01

**Authors:** Rodica Padureanu, Carmen Valeria Albu, Radu Razvan Mititelu, Manuela Violeta Bacanoiu, Anca Oana Docea, Daniela Calina, Vlad Padureanu, Gabriela Olaru, Raluca Elena Sandu, Ramona Denise Malin, Ana-Maria Buga

**Affiliations:** 1 Department of Biochemistry, University of Medicine and Pharmacy of Craiova, 200349 Craiova, Romania; zegheanurodica@yahoo.com (R.P.); razvanmititelu@rocketmail.com (R.R.M.); raluv_2005@yahoo.com (R.E.S.); 2 Department of Neurology, University of Medicine and Pharmacy of Craiova, 200349 Craiova, Romania; carmenvaleriaalbu@yahoo.com (C.V.A.); ramona_denise@yahoo.com (R.D.M.); 3 Department of Physical Therapy and Sports Medicine, University of Craiova, 200207 Craiova, Romania; manuela_bacanoiu@yahoo.com (M.V.B.); olarugabriela246@yahoo.com (G.O.); 4 Department of Laboratory Medicine, County Clinical Emergency Hospital of Craiova, 200642 Craiova, Romania; 5 Department of Toxicology, University of Medicine and Pharmacy of Craiova, 200349 Craiova, Romania; daoana00@gmail.com; 6 Department of Clinical Pharmacy, University of Medicine and Pharmacy of Craiova, 200349 Craiova, Romania; calinadaniela@gmail.com; 7 Department of Internal Medicine, University of Medicine and Pharmacy of Craiova, 200349 Craiova, Romania; vldpadureanu@yahoo.com

**Keywords:** multiple sclerosis, oxidative stress, neurodegeneration

## Abstract

The study aims to explore the oxidative status related to inflammation in peripheral blood of stable relapsing-remitting multiple sclerosis (MS) patients with low disability. In this study, 31 people were included and divided into two groups: an MS group in which 16 relapsing-remitting MS patients with a low disability level (age 38.9 ± 7.08, EDSS median 2.5) were included and a control group that contains 15 healthy volunteers of similar age to the MS group. Thiobarbituric acid reactive substances (TBARS), protein carbonyl level (PCO), total antioxidant capacity (TAC) as oxidative stress markers, neutrophil/lymphocyte ratio (NLR), and erythrocyte sedimentation rate (ESR) were analyzed in the peripheral blood sample of the healthy and the MS patients to establish the oxidative stress/inflammatory level using conventional plasma markers. In this study, we showed that the pro-inflammatory status of the relapse-remitting stage of diseases can be easily and accurately appreciated by NLR. An increased NLR is associated with a decreased antioxidant capacity, even in the early stage of neuronal damage. Oxidative stress associated with inflammation aggravates the functional outcome, potentiates neuronal damage, and can accelerate the progression of the disease.

## 1. Introduction

Oxidative stress involves a key pathway that contributes to many pathological processes including Multiple Sclerosis (MS) [1,2]. A balance between reactive oxygen species (ROS) production and antioxidant defence is crucial to prevent any structural damage to the CNS. The research studies in the last decade were focused on ROS action in CNS and its correlation with normal and pathological signaling pathways that target the neuronal cells [3,4].

It is well known that ROS production is necessary for neuronal cell stability, modulation of synaptic plasticity and brain functions (e.g., apoptosis, synaptic plasticity-related pathway including cognition, immune system) but the border between necessary and harmful processes is still very sensitive [5,6].

An increased incidence of neurological and neurodegenerative diseases with aging can be a consequence of an imbalance between ROS production and antioxidant capacity of the brain tissue [7,8]. When the level of ROS increases in the cells, the capacity of the body to produce antioxidant molecules in a sufficient proportion is crucial to prevent the oxidative stress damage [9,10].

Many pathological conditions of the CNS are associated with increased oxidative stress (e.g., Parkinson’s disease, Alzheimer’s disease) [11,12]. The pathophysiology of MS is still under debate. Many factors are potentially involved in the MS progression, including oxidative stress that promotes neurovascular unit injury and induces a pro-inflammatory state [13,14]. 

In the early stage of MS, the immune system hyperactivation is the main pathological feature that promotes extensive damage of myelin by increasing the release of ROS. Later, in the chronic phase of MS, the main scenario involves structural and functional damage as a result of oxidative stress [15].

The neuronal tissue damage is a result of the inadaptive response of the antioxidant pathway in the early phase of the disease. MS progression can have a different clinical course related to the variability of adaptive mechanisms (relapsing, remitting or primary progressive courses). In this light, our hypothesis is that the disease progression can be decided in the early stages by promoting an enhanced and sustained antioxidant capacity of CNS tissue as early as possible. Furthermore, oxidative stress is associated with immune system hyperactivation through microglial cell activation. The activated microglial cells can release pro-oxidative signaling molecules (e.g., interleukin, cytokine) that can modulate the immune pathway, metabolic and/or apoptotic pathway in a time-dependent manner [9]. 

Since oxidative stress and inflammation correlate in a vicious cycle, recent studies have identified the biomarkers of oxidative stress and antioxidant capacity as potential tools to assess the inflammatory status in Multiple Sclerosis (MS) patients [16,17,18].

The aim of this study was to find potential peripheral biomarkers to show oxidative stress levels and antioxidant capacity in peripheral blood samples collected from MS patients in the remitting stage. Thiobarbituric acid reactive substances (TBARS), protein carbonyl level (PCO) and total antioxidant capacity (TAC) were analyzed in the peripheral blood sample of the healthy and MS patients with a low disability level (EDSS ≤ 4) to prove the oxidative stress level using conventional plasma markers. Also, our aim was to analyze the correlation between oxidative stress markers and inflammation in the subclinical stage of neuronal damage.

## 2. Materials and Methods

### 2.1. Ethical Issues

The study was approved by local institutional ethics committees of the University of Medicine and Pharmacy of Craiova (Registration No. 96) according to European Union Guidelines (Declaration of Helsinki). All participants gave written informed consent to be included in the study. Forty-one adult subjects were included in the study, 16 relapsing-remitting MS subjects (RRMS) (age 38.9 ± 7.08; first 10 years of disease progression and EDSS ≤ 4), 10 secondary progressive MS subjects (SPMS) (more than eight years of disease progression and EDSS > 2) and 15 adult healthy subjects with an appropriate median of age (age 37.1 ± 11.2). Exclusion criteria were the following: pregnancy, drug abuse, comorbidities that could increase the systemic inflammation (e.g., diabetes, metabolic syndrome), corticoids or non-steroidal anti-inflammatory drugs. All patients included in the MS group are non-smokers and received the first line-therapy (Glatiramel acetate or interferon beta).

### 2.2. Sample Collection and Handling

The blood samples were collected in the morning after 12 h of fasting by an experimented phlebotomist using EDTA (ethylene-diamine-tetraacetic acid) tube collection, in order to prevent blood clotting. Plasma and red blood cell (RBC) fractions were separated by centrifugation at 3600 rpm at 4 °C for 10 min (5417R Eppendorf centrifuge). The sediments were divided from plasma immediately after centrifugation and oxidative stress markers were analyzed in the plasma.

### 2.3. Thiobarbituric Acid Reactive Substances Assay

To analyze the lipid peroxidation level, we performed the thiobarbituric acid reactive substances (TBARS) plasma analysis using a UV spectrophotometric method [19,20]. The principles of procedure: malondialdehyde (MDA) is a common marker used to establish the oxidative stress index in human plasma. We analyzed the lipid peroxidation level by quantifying MDA concentration from deproteinized human plasma. Briefly, 0.1 mL human plasma was treated with 5% trichloroacetic acid (TCA) and 0.2 M Tris-HCl pH = 4.7 (*v*/*v*). After 10 min of incubation at room temperature (RT) the sample was mixed with 1 mL of 0.55 M thiobarbituric acid (TBA) in 2 M sodium sulphate, heated at 90 °C for 45 min and cooled down in ice [20,21]. The optical density (OD) of the sample was measured at 530 nm using a UV-VIS spectrophotometer. The TBARS concentration was calculated using the molar extinction coefficient of MDA (1.55 × 105 M^−1^ cm^−1^). The results were expressed as TBARS (µmol/L).

### 2.4. Protein Carbonyl Assay

Protein carbonyls may be generated by irreversible oxidation of several amino acid side chains (Lysine, Arginine, Threonine, and Proline) from the carbonylation site in protein structure or by increased advanced glycation end products (AGE) production. The current successful method used to assess protein carbonyl (PCARB) concentration is a spectrophotometric assay using 2,4-dinitrophenylhydrazine (DNPH) [20,22,23]. The human plasma samples were mixed with 20% TCA (*v*/*v*), incubated for 15 min on ice and separated by centrifugation for 5 min/ 12,000 rpm at 4 °C. After centrifugation, the supernatant was discarded and the pellet was treated with 0.5 mL 10 mM 2,4-dinitrophenylhydrazine (DNPH) in 2.5 M HCl for the test sample. The samples were incubated in the darkroom for 1 h with intermittent shaking every 15 min. After incubation time, the samples were separated by centrifugation for 5 min at 12,000 rpm at 4 °C and 10% TCA was added on the pellet, stored in ice for 10 min, mixed by vortexing and separated by centrifugation for 5 min at 12,000 rpm at 4 °C. The supernatant was removed and two washing steps were performed with 1 mL ethanol: ethyl acetate (1:1, *v*/*v*) to remove the excess of DNPH. The protein pellet was solved in 1 mL of 5M urea (pH = 2.3) at 37 °C for 10 min and separated by centrifugation for 5 min at 12,000 rpm at 4 °C. Finally, the OD samples were measured at λ = 375 nm using a UV-VIS spectrophotometer. The PCARB content was calculated based on the molar extinction factor of DNFH (22,000 M^−1^ cm^−1^). The control sample with 2.5 M HCL was used during the protocol. The PCARB concentration was expressed as nmol/mg of protein. Total protein concentration in the sample was assessed using Bradford reagent [24]. 

### 2.5. Total Antioxidant Capacity (TAC) Assay

TAC is usually used to assess the antioxidant status in human samples associated with diseases. TAC assessment show as a general capacity of the body to fight against oxidative stress by realizing antioxidant compounds. This capacity can be easily assessed in human plasma using a spectrophotometric method [20,25]. The human plasma samples diluted 1: 25 in 1×Phosphate Buffer Saline (PBS, pH = 7.4) were mixed with 0.1 mM 2,2 diphenyl-1-picrylhydrazyl reagent (*v*/*v*) and incubated in a dark room for 30 min. After incubation time, the samples were separated by centrifugation for 3 min at 14,000 rpm and OD was read at λ = 520 nm using a UV-VIS spectrophotometer. TAC was expressed as mmol DPPH/L.

### 2.6. Neutrophil/Lymphocyte Ratio 

Neutrophil and lymphocyte were counted in peripheral blood obtained by standard venipuncture (EDTA collection tube) using an automatic flow cytometry analyzer (CELL-DYN Ruby System, Abbott Diagnostics). The neutrophil/lymphocyte ratio (NLR) was calculated by dividing the neutrophil number by lymphocyte number [26]. 

### 2.7. Statistical Analysis

Data were analyzed using GraphPad Prism 5.0 software. The comparison of oxidative stress markers between groups was performed using unpaired non-parametric Mann–Whitney *t*-test. Non-parametric Pearson’s was calculated to test the correlation between biochemical and clinical variables (EDSS). *p* values under 0.05 (*p* ≤ 0.05) were selected as significant changes. The data are calculated as mean ± standard error of the mean (SEM).

## 3. Results

### 3.1. Clinical and Biological Characteristics

The MS patients included in this study are in the relapsing-remitting stage of diseases (MS group 1) with a low disability level (EDSS = 1–4) and secondary progressive MS subjects (more than eight years of disease progression and EDSS ≥ 2). The differences between the MS groups’ and the healthy control group’s biological variables, age, disability score (EDSS) are presented in Table 1.

Hyperactivation of the immune system response can be monitored using a blood test marker like erythrocyte sedimentation rate (ESR) and neutrophil/lymphocyte ratio (NLR) (Table 2). 

### 3.2. Clinical and Biological Characteristics

#### 3.2.1. Neurophil/Lymphocyte Ratio 

**Figure 1:**
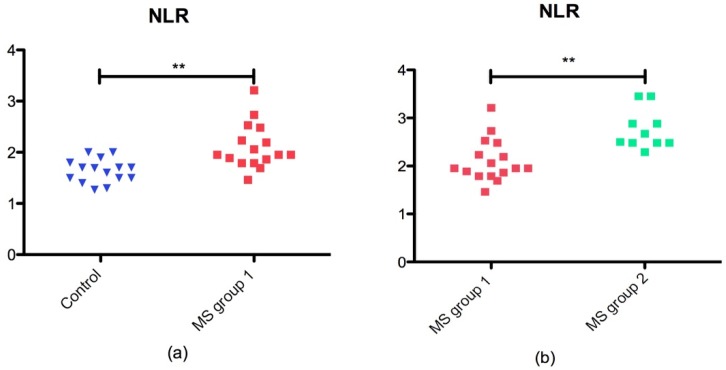
NLR in MS groups and control group: (**a**) scatter plot of NLR in control group and MS group 1 (*p* = 0.0025 **); (**b**) scatter plot of NLR in MS group 1 and MS group 2 (*p* = 0.0010 **).

Currently, NLR was described as a new biomarker associated with inflammation that is accurate and easy to perform. NLR was significantly higher in MS group 1 than in the control group (*p* = 0.0025) as presented in Figure 1a. Also, we found that NLR was significant higher in MS group 2 vs MS group 1 (*p* = 0.001) as shown in Figure 1b.

#### 3.2.2. Oxidative Stress Markers vs. Total Antioxidant Capacity (TAC) in MS Patients

**Figure 2:**
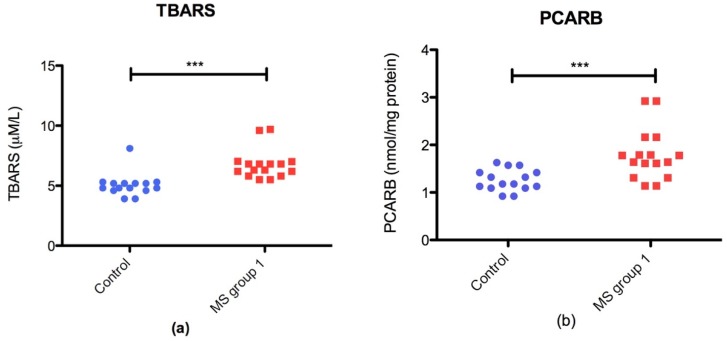
Plasmatic oxidative stress markers in RRMS groupvs control group: (**a**) scatter plot of TBARS levels; (**b**) scatter plot of PCARB (protein carbonyl) level in control group and RRMS group.

The oxidative stress marker in the plasma was significantly increased in RRMS patients compared with the control group. TBARS (*p* = 0.0001 ***) and PCARB (*p* = 0.0007 ***) were significantly higher in MS patients than those in the control group (Figure 2)

**Figure 3:**
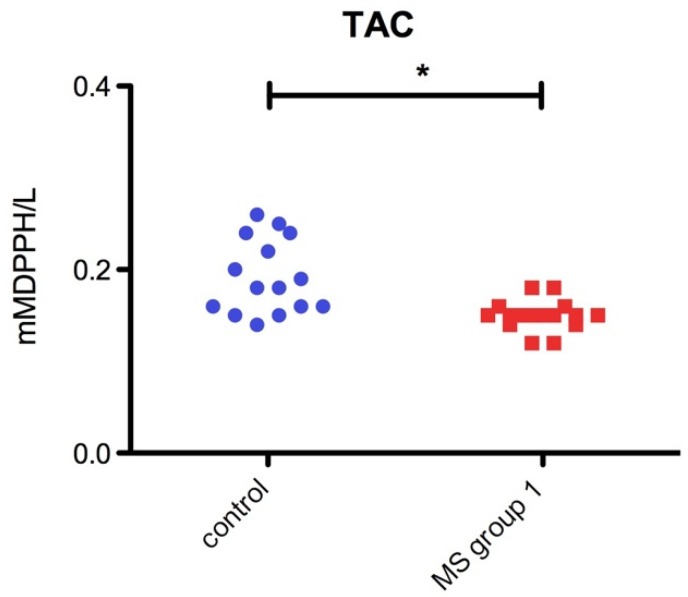
Bar plot (mean with SEM) of plasmatic TAC level in Control group and RRMS group (*p* = 0.04 *).

In contrast, total antioxidant capacity (TAC) was significantly decreased (*p* = 0.04 *) in RRMS patients as compared to the healthy control group (Figure 3).

#### 3.2.3. Correlation of Oxidative Stress Status with Disability Stage (EDSS) and Inflammatory Status

**Figure 4:**
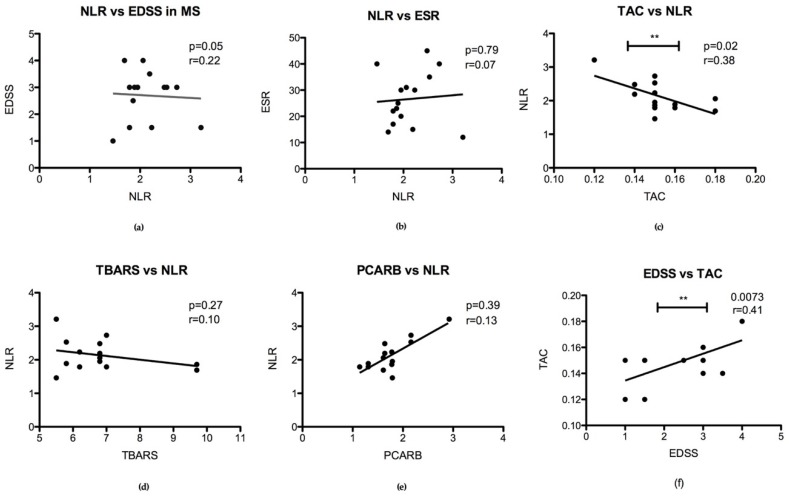
Correlation between inflammation marker NLR, oxidative stress status in plasma and disability stage (EDSS): (**a**) NLR correlation with EDSS (*p* = 0.05); (**b**) correlation between NLR and ESR (*p* = 0.79); (**c**) TAC and NLR correlation (*p* = 0.02 **); (**d**) TBARS and NLR correlation (*p* = 0.27); (**e**) PCARB with NLR correlation (*p* = 0.39); (**f**) EDSS and TAC correlation in MS group 2 (*p* = 0.0073 **).

Interestingly, we found a significant negative correlation between TAC level and NLR (*p* = 0.02) in group 1 MS patients’ plasma (Figure 4c) and between EDSS and TAC in group 2 MS patients (Figure 4f). We did not find any significant correlation between EDSS and NLR (*p* = 0.05) or ESR and NLR (*p* = 0.79) in MS patients groups. Also, oxidative stress markers (TBARS and PCARB) did not correlate significantly with NLR in the MS groups (Figure 4).

Also, we did not find a significant correlation of EDSS with PCARB and TBARS levels in MS group 2 patients.

#### 3.2.4. Receiver Operating Characteristic Curve (ROC Analysis)

**Figure 5:**
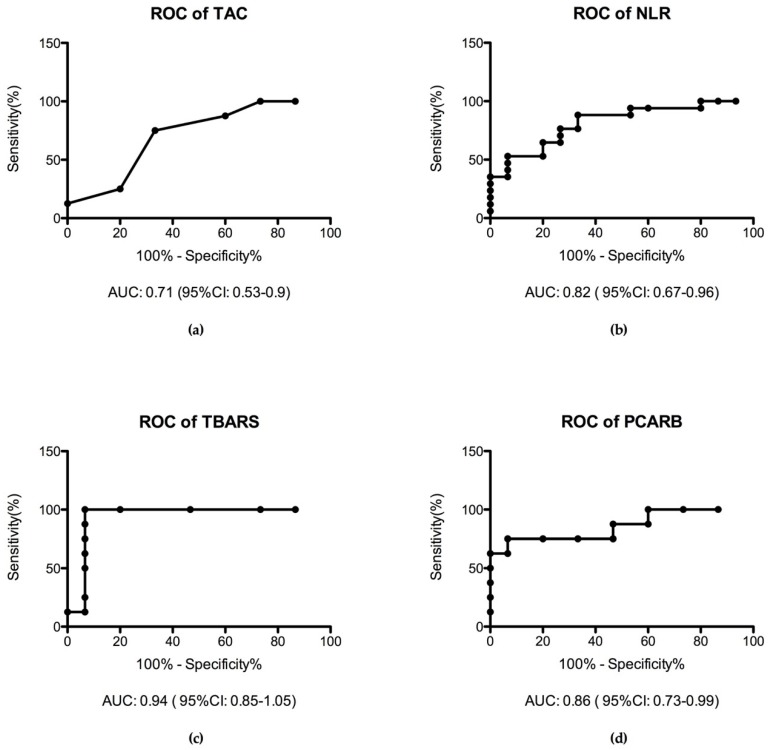
ROC analysis of inflammation marker NLR and oxidative stress marker in plasma: (**a**) TAC assay characteristics in MS (AUC = 0.71); (**b**) NLR characteristics (AUC = 0.82); (**c**) TBARS specificity (AUC = 0.94); (**d**) PCARB assay performance (AUC = 0.86).

We performed a receiver operating characteristic (ROC) analysis of the inflammation marker and the oxidative stress marker in the MS patient groups compared with the healthy control group. Interestingly, we found TBARS as the most specific marker of oxidative stress with the highest area under the curve (AUC) value (AUC = 0.94) followed by PCARB level (AUC = 0.86). NLR has a moderate specificity (AUC = 0.82) to MS diseases in the early stage. In contrast, TAC has low specificity (AUC = 0.71) in this stage of diseases (Figure 5). 

Being a graphical plot that illustrates the diagnostic ability of a binary classifier system, the ROC curve is actually the sensitivity as a function of fall-out. Considering the diagnosis as the output and any different parameter as the single input for the classifier we can use the area under the ROC curve as a measure for the ration between the parameter taken into consideration and the diagnosis.

A good interpretation of the graphical representation uses the following rule: one point in space is better than another if it is situated to the northwest of the square. Technically, if the true positive rate is high and the false positive rate is low then the prediction is better. We can see from Figure 5, that the points are above the line of no-discrimination implying a good classification. In terms of the value of the AUC we can see that for TBARS the classification is very good, AUC = 0.94, for the NLR and PCARB the classification is good, AUC = 0.82, and AUC = 0.86 respectively, whereas for the TAC the classification is fair, AUC = 0.71.

## 4. Discussion

To our knowledge, this is the first study to evaluate the NLR and oxidative stress association in MS patients. In this study, we found that the MS patients display a significant increase of oxidative stress markers in plasma (TBARS and PCARB) suggesting that oxidative stress induction started in the RRMS stage with a low level of irreversible neuronal damage. This induction is due to an imbalance between oxidative stress triggers and the capacity of the body to neutralize ROS as shown by a significant decrease of the TAC assay in plasma of MS patients. In the present work, we employed a TAC assay in plasma, to test the general oxidative stress protection capacity of the body in response to an increased ROS level. TAC includes all antioxidant compounds (enzyme or other molecules) that protect the body, not only at the systemic level but also CNS level [27,28,29]. 

Oxidative stress affects endogenous antioxidant defense capacity leading to neuronal damage [30,31]. Also, endogenous sources of ROS include inflammation, and ROS can act as a secondary brain injury after long-term pro-inflammatory status in a vicious cycle. In line with other research studies [18,32], we found a decreased antioxidant capacity that exposes the brain tissue to neuronal damage. Also, we showed that the TAC marker in plasma is significantly correlated with the inflammation marker NLR in RRMS patients and can be useful in the clinical management of diseases. There are still controversial findings of oxidative stress involvement in neuronal damage in the RRMS stage of diseases. Some studies suggest the importance of ROS action in the progressive stage of MS, and not in the pro-inflammatory stage [15,33]. These studies prove that the antioxidant defense system is affected in RRMS and is correlated with inflammatory status. Also, we found a low specificity of TAC assay alone in RRMS diseases. In contrast, TAC is significantly correlated with EDSS in secondary progressive MS patients. 

NLR was described before to be an independent predictive marker of MS disease activity [26]. Studies before proved that the NLR is correlated with poor outcomes in many other diseases (e.g. acute myocardial infarction, cancers, autoimmune diseases, etc.) [34]. Also, neutrophils granule contains enzyme-like lysozyme or lactoferrin which can be markers of organ-specific inflammation [35]. However, only limited studies described NLR association in MS diseases [36,37] and it is still a growing interest aiming to establish potential serological markers associated with a local and systemic inflammatory status that are cost-effective and minimally invasive. We found that NLR did not correlate with EDSS and has a medium specificity alone in MS diseases, but these findings should be validated on a large cohort. 

The brain tissue is highly sensitive to ROS action due to the increased consumption of oxygen and a high concentration of polyunsaturated fatty acids that are sensitive to lipid peroxidation [29,38]. In this light, the lipid peroxidation indicator in plasma was assessed using the detection of TBARS released by oxidative processes of polyunsaturated fatty acids (PUFA). An increased level of TBARS was found in RRMS patients but did not correlate with inflammation status. Interestingly, the TBARS assay displayed the highest specificity in the RRMS and can be a useful tool in MS disease management. In contrast, we found a significant correlation of EDSS with TAC in SPMS patients and we did not find a significant correlation with PCARB and TBARS in the stage of this disease. A possible explanation is due to the limited number of patients included in this study. 

In order to support these findings, another oxidative stress marker PCARB was significantly increased in MS patients. Increased PCARB level was described before in neurodegenerative diseases but the link between PCARB level, inflammation and disease outcome is still under debate. PCARB is a useful tool to assess the irreversible changes in protein structure and considered a hallmark of increased ROS action and maybe a key player in MS disease progression [39,40]. In this study, we found that PCARB has an increased specificity (after TBARS) in MS diseases and did not correlate with NLR.

## 5. Conclusions

Enhanced protection of CNS by a sustained antioxidant capacity plays a crucial role in MS patient management to limit the irreversible neuronal damage. An increased level of oxidative stress markers and decreased level of TAC starting from subclinical neuronal damage was found. Also, in this study we showed that: (1) the pro-inflammatory status of the relapse-remitting stage of diseases can be easily and accurately appreciated by NLR and (2) an increased NLR is associated with a decreased antioxidant capacity, even in the subclinical stage of neuronal damage that affects the functional outcome (EDSS = 2.5), potentiates neuronal damage and can accelerate the progression of the disease; (3) TBARS assay displayed the highest specificity in the RRMS patients and can be a useful tool in MS diseases management.

These show that oxidative stress is a key factor, together with the inflammatory state, that can contribute to disease progression. An increased oxidative stress level with a proinflammatory status can be a preclinical sign applying for proper intervention to support the antioxidant homeostasis. The NLR is an easy-to-perform biomarker that is accurate and can show the proinflammatory status of MS patients. In our opinion, a multimodal approach is needed to be added to MS management starting in the first 10 years of disease progression in order to improve the functional outcome. However, our study is limited to a small number of patients and has to be further explored in a large cohort. 

## Figures and Tables

**Table 1:** Demographic data of multiple sclerosis (MS) and healthy control group.

Groups	Age Mean ± SD Age Range	Sex	Disease Progression Mean (Range)	EDSS Mean
MS group 1 ^1^	38.9 ± 7.08 (28–50)	13 Female 3 Male	7 years (0–10)	2.5 (1–4)
MS group 2 ^2^	54 ± 9.23 (47–69)	10 Female	13 years (8–21)	5 (2–7)
Control group	37.1 ± 11.2 (26–49)	12 Female 3 Male	-	-

^1^ RRMS (relapsing-remitting multiple sclerosis) group with low disability level (EDSS = 1–4); ^2^ SPMS (secondary progressive multiple sclerosis) group (EDSS = 2–4).

**Table 2:** Biological markers of inflammation status in MS and healthy control groups.

Groups	ESR Mean ± SD	NLR Mean ± SD
MS group 1	24.5 ± 9.15 ^1^	2.11 ± 0.11 ^1^
MS group 2	35.4 ±16.5	2.8 ± 0.71 ^1^
Control group	12.79 ± 1.84	1.61 ± 0.08

^1^ These parameters were significant higher.
